# Supplementary material for: Prevalence and predictors of depression, anxiety and stress among frontline healthcare workers at COVID-19 isolation sites in Gaborone, Botswana
Source: PLoS One. 2022 Aug 23;17(8):e0273052. doi: 10.1371/journal.pone.0273052 (PMC9398030; doi:10.1371/journal.pone.0273052)
Supplement: Attachment — Submitted filename: Response to Reviewers.docx [file pone.0273052.s002.docx]

**Response to Reviewers**

**1. Abstract in the results for both the prevalence and the OR please add their confidence intervals.**

Confidence intervals have been added for both the prevalence and odds ratios.

**2. Line 111/112 - what is this exhaustive sampling.**

The phrase “exhaustive sampling” has been removed and is replaced with a statement of how the sampling was done which is that all consenting eligible participants were enrolled.

**3. Lines 120/121 - There is a little language issue. Usually, when we do not know where the real proportion would be we choose the proportion of 0.5 because this is where the proportion gets the largest variance i.e the largest sample size for a certain margin of error.**

This has been corrected. It now reads “Based on previous studies, the prevalence was expected to fall between 10% and 90%. The precision was set at 5% or 0.05. A P of 0.5 was chosen to achieve the largest sample size for the specified margin of error. The calculated sample size was 385 participants”

**4. Line 140: it seems it should be depression, anxiety and stress. Correct, please.**

This has been corrected.

**5. Lines 142 to 147: was this tool tested in Gaberone/Botswana previously?**

To the best of our knowledge, the tool has not been tested in Botswana. It has however been validated in other Sub Saharan countries. We tested the tool through a pilot. We have described this pilot in the paper.

We have cited a systematic review that reports use of this tool in other Sub-Saharan countries.

**6. Somewhere in the data analysis section, we miss a clear indication of how you did dichotomize the scales. In fact, we do not know how you did classify each person into each of the 6 levels (like in table 3), to begin with.**

We have now described in detail how the different levels of the 3 domains were calculated in the analysis section. The statement reads, “Participants were categorized into normal, mild, moderate, severe and extremely severe according to the cut-off in the respective subscales. The depression subscale is made up of questions 3,5,10,13,16,17,21,24,26,31,34,37,38 and 42. This scale was divided into normal (0-9), mild depression (10-13), moderate depression (14-20), severe depression (21-27) and extremely severe depression (>28). The anxiety subscale is made of questions 2,4,7,9,15,19,20,23,25,28,30,36,40 and 41. The scale was divided into normal (0-7), mild anxiety (8-9), moderate anxiety (10-14), severe anxiety (15-19) and extremely severe anxiety (>20). The rest of the questions make up the stress subscale. The scale was divided into normal (0-14), mild stress (15-18), moderate stress (19-25), Severe stress (26-33) and extremely severe stress (>34).

**7. Table 3: I would suggest adding confidence intervals for the prevalence at least for the abnormal row.**

The confidence intervals have been added for all the proportions in table 3.

**8. Put below the table the abbreviations and the name of the test used**

This has been done

**9. Sadly, again, we use inferior methods to analyse ordinal variables (depression, anxiety and stress levels). For example, the Chi-square applied in table 4 just checks whether there is one cell particularly different from the expected or not. Such a test discards the fact that the depression level has an order, and we are not informed what is the direction of an association if it exists. This is just a lament. I am not requesting the authors to change the analysis.**

We have removed the p values as per recommendation by the reviewer. (see below)

**If there are no restrictions, please upload the minimal anonymized data set necessary to replicate your study findings as either Supporting Information files or to a stable, public repository and provide us with the relevant URLs, DOIs, or accession numbers.**

We have uploaded the dataset as supporting information.

**Reviewer #1:**

**1.Line 103:The second sentence in the paragraph is not important as in the next paragraph the participants have been defined**

The sentence has been removed

**2.Line 111: I would avoid using the phrase the sampling was exhaustive but rather describing what was done to get an exhaustive sampling**

The phrase was removed

**3.line 114 sample size calculation: Not clear why the authors decided to use a range for expected prevalence. Though I imagined the authors have obtained a good sample size, I would have advised using a prevalence from a previous study that was done in a geographical location closest to the study site.**

We used a P of 0.5 or 50% to get the maximum sample size for the specified margin of error. The calculated sample size is adequate and higher than it would be with a known prevalence.

**Table 4: I am sure the importance of the p value in the table 4, this is purely a descriptive statistics of the participants.**

We have removed the p value in table 4.
